# Response to SARS-CoV-2 initial series and additional dose vaccine in pediatric patients with predominantly antibody deficiency

**DOI:** 10.3389/fimmu.2023.1217718

**Published:** 2023-07-27

**Authors:** Megha Tandon, Daniel V. DiGiacomo, Baijun Zhou, Paul Hesterberg, Chen E. Rosenberg, Sara Barmettler, Jocelyn R. Farmer

**Affiliations:** ^1^ Division of Rheumatology, Allergy and Immunology, Department of Medicine, Massachusetts General Hospital, Boston, MA, United States; ^2^ Harvard Medical School, Boston, MA, United States; ^3^ Division of Allergy and Inflammation, Beth Israel Lahey Health, Boston, Massachusetts, United States

**Keywords:** COVID-19, vaccine, predominantly antibody deficiency, SARS-CoV-2, inborn error of immunity, primary immune deficiency

## Abstract

Data regarding response to SARS-CoV-2 immunization in pediatric patients with predominantly antibody deficiency (PAD) is limited. We evaluated SARS-CoV-2 immunization response by anti-SARS-CoV-2-spike antibody level in 15 pediatric PAD patients. These data were compared to a published cohort of adult PAD patients (n=62) previously analyzed following SARS-CoV-2 immunization at our single center institution. We evaluated demographics, clinical characteristics, immunophenotype, infection history, and past medication use by chart review. Following a two-dose monovalent initial series SARS-CoV-2 immunization, mean anti-SARS-CoV-2-spike antibody levels were significantly higher in pediatric PAD patients compared to adult PAD patients (2,890.7 vs. 140.1 U/mL; p<0.0001). Pediatric PAD patients with low class-switched memory B-cells, defined as <2% of total CD19+ B-cells, had significantly lower mean anti-SARS-CoV-2-spike antibody levels than those without (p=0.02). Following a third-dose monovalent SARS-CoV-2 immunization, the mean anti-SARS-CoV-2-spike antibody levels in pediatric PAD patients significantly increased (2,890.7 to 18,267.2 U/mL; p<0.0001). These data support Centers for Disease Control guidelines regarding three-part SARS-CoV-2 vaccine series, including in the pediatric PAD patient demographic.

## Introduction

Predominantly antibody deficiency (PAD) is the most commonly diagnosed inborn error of immunity (IEI) and the most frequent symptomatic primary immunodeficiency disorder world-wide. PAD encompasses a heterogeneous collection of disorders characterized by increased susceptibility to infections, low immunoglobulin levels, and impaired vaccine responses (1). The clinical presentation can be variable, ranging from mild symptoms to severe complications. Patients with PAD are at risk of ongoing disease progression, development of autoimmunity, end-organ damage, and reduced life expectancy (2).

Studies have demonstrated that children produce long-term antibody responses to COVID-19 infection (3). Healthy children are less likely to have severe COVID-19 compared to adults (4, 5). Multiple studies have demonstrated worse COVID-19 infection outcomes and more severe disease in children who had received immunosuppressant medication or had an immunocompromising condition such as cancer compared to healthy children (6–8).

There have been several studies investigating COVID-19 infection outcomes in patients with IEI. A review of the Italian Primary Immunodeficiency Network found that there were no deaths among 33 COVID-19 positive children with an underlying IEI condition (9). Abolhassani et al. found that among 381 published cases of COVID-19 infection in pediatric IEI patients, 23.6% of them had severe COVID-19, with an overall mortality rate of 8.7% (10).

The trials evaluating the safety and effectiveness of the SARS-CoV-2 vaccine in children and adolescents excluded those who had immunodeficiency diseases including PAD (11, 12). Subsequent evaluation of the effectiveness of the SARS-CoV-2 vaccine in adult primary immune deficiency patients found lower humoral immune responses in adult PAD patients when compared to healthy controls (13–15).

To date, most studies regarding the efficacy of the SARS-CoV-2 vaccine in immunocompromised children are limited to those receiving immunosuppressants for a medical condition such as renal disease, malignancy, or organ transplant (16, 17). Among IEI patients, Erra et al. described the safety and efficacy of a two-dose mRNA vaccine series in adolescent IEI patients ages 12 years and older and showed that SARS-CoV-2 vaccine immunogenicity after a two-dose series was lower in pediatric IEI patients when compared to healthy controls (18). However, there is limited data regarding efficacy of a third dose of the monovalent SARS-CoV-2 vaccine in pediatric patients with PAD.

The aim of this study was to assess the efficacy of the SARS-CoV-2 vaccine, following two-dose initial series and third-dose boost, in pediatric patients of all ages with PAD.

## Methods

This study was performed at Mass General Brigham under an institutional review board–approved protocol (No. 2021P002414). Antibody response to the SARS-CoV-2 vaccine in patients with known PAD was evaluated. Pediatric PAD patients who received an initial two-dose SARS-CoV-2 vaccine series between May 2021 and September 2022 were included. The PAD diagnoses were confirmed by manual chart review by a clinical immunologist and met consensus definitions for PAD (19–21). Patients with primary PAD were further subclassified as mild (including IgG subclass deficiency, specific antibody deficiency (SAD), and primary hypogammaglobulinemia), moderate (including uncomplicated common variable immune deficiency (CVID), defined as an absence of co-occurring autoinflammatory clinical features), and severe (including complicated CVID/SAD, defined as the presence of concomitant autoinflammatory clinical features) (22). Patients with confounding variables at the time of immunodeficiency diagnosis (e.g., ongoing immunosuppression without the potential for discontinuation) were considered as secondary PAD.

We evaluated demographic information and clinical characteristics including the type of PAD, vaccine type received, and prior immunophenotyping, including native immunoglobulin levels, native vaccine titers (e.g., pneumococcal polysaccharide [PPSV23]; Hemophilus influenza B [HIB], and tetanus, diphtheria, and pertussis), and peripheral blood lymphocyte counts as available. We evaluated previous and current use of immunoglobulin replacement and other immunosuppressants or biologics received in the past or in close proximity to vaccination (defined as 6 months before to 1 month after immunization). We also evaluated infection history, outcome, treatment, and tixagevimab/cilgavimab use.

Serologic assays were performed using the Roche (Basel, Switzerland) Elecsys Anti-SARS-CoV-2 S-antibody test (evaluating antibodies to the SARS-CoV-2 spike (S) protein receptor binding domain; anti-spike antibody). This test is semiquantitative and has been correlated with neutralizing immunity (23, 24). The Roche S-antibody assay reports in absorbance units per milliliter with values of 0.8 U/mL or greater considered reactive (25).

We used the Student t-test to compare mean anti-spike antibody values and the Mann-Whitney U-Test for comparing median values. All antibody responses to SARS-CoV-2 vaccine were reported as geometric means (95% confidence interval [CI]). Statistical analyses were completed with SAS software (version 9.4, SAS Institute, Cary, NC) and Prism software (version 7.01, Reston, Va); two-tailed P-value less than.05 was considered significant.

## Results

### Pediatric PAD patient characteristics

15 pediatric patients with PAD were included (**Table 1**). The mean age was 10.7 years (range 4-16 years); the majority of patients were male (93.3%) and non-Hispanic white (80%). All patients included in this cohort received the monovalent BNT162b2 (Pfizer-BioNTech) SARS-CoV-2 vaccine. Six patients were classified as mild PAD (40%), three moderate PAD (20%), four severe PAD (27%), and two had secondary PAD (13%). Eight patients were on immunoglobulin replacement therapy (53.3%), with seven receiving subcutaneous immunoglobulin (SCIG) replacement and one receiving intravenous immunoglobulin (IVIG) replacement. None of these patients received tixagevimab/cilgavimab or other monoclonal anti-SARS-CoV-2 antibodies during the duration of the study.

**Table 1:** Demographic characteristics of participants.

	Pediatric PAD (n=15)	Adult PAD (n=62)
Age (years, mean (range))	10.7 (4-16)	52.5 (20-72)
Gender (n, %M)	14, 93.3%	19, 30.7%
Non-Hispanic White (n, %)	12, 80.0%	59, 95%
Vaccine, type (n, %)		
BNT162b2 (Pfizer) <5 years	1, 6.7%	–
BNT162b2 (Pfizer) 5-11 years	7, 46.7%	–
BNT162b2 (Pfizer) 12+ years	7, 46.7%	–
Vaccine, cumulative doses received (n, %)		
1	–	–
2	4, 26.7%	62, 100.0%
3	9, 60.0%	–
4	2, 13.3%	–
Days from last vaccine to draw, median		
Dose 2	85	31
Dose 3	118.5	–
PAD Severity (n, %)		
Mild	6, 40.0%	12, 23.1%
Moderate	3, 20.0%	21, 40.4%
Severe	4, 26.7%	19, 36.5%
PAD Type (n, %)		
Primary Hypogammaglobulinemia	1, 6.7%	4, 7.7%
Specific Antibody Deficiency	1, 6.7%	5, 9.6%
IgG Subclass Deficiency	4, 26.7%	3, 5.8%
Common Variable Immune Deficiency	3, 20.0%	21, 40.4%
Complicated Predominantly Antibody Deficiency	4, 26.7%	19, 36.5%
Secondary Hypogammaglobulinemia	2, 13.3%	10, 16.1%
Current Immunoglobulin Replacement Therapy (n, %)		
IVIG	1, 6.7%	19, 30.6%
SCIG	7, 46.7%	24, 38.7%
Naturally acquired COVID-19 infection (n, %)	8, 53.3%	–

SARS-CoV-2 antibody levels were measured after pediatric PAD patients received their initial two-dose SARS-CoV-2 vaccine series (n=13), and after the third-dose vaccination (n=8). Median time from vaccination to SARS-CoV-2 antibody level testing was 85 days from initial series and 119 days from third-dose vaccine for pediatric PAD patients. In contrast, median time from vaccination to SARS-CoV-2 antibody level testing for adult PAD patients was 31 days from initial series, which was a statistically shorter time interval when compared to the pediatric cohort (p=0.0002). Two of 15 PAD patients had SARS-CoV-2 infections after their third-dose booster, but before their blood draw. These patients had SARS-CoV-2 antibody level testing performed after they cleared their infections (at a median time of 71.5 days post-positive polymerase chain reaction [PCR] test) (**Supplemental Figure 1**).

### SARS-CoV-2 vaccine response is higher in pediatric compared to adult PAD patients

**Figure 1:**
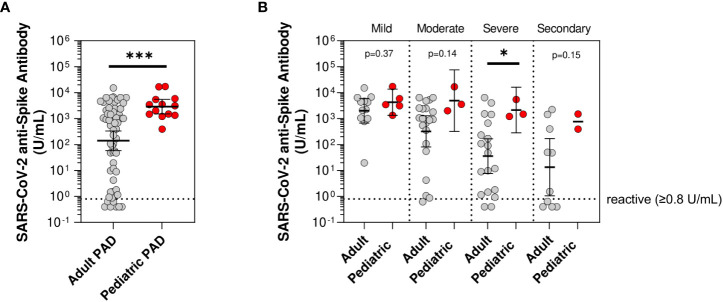
Mean anti-spike antibody levels in pediatric compared to adult predominantly antibody deficiency (PAD) patients. SARS-CoV-2 anti-spike antibody levels (U/mL) following initial series two-dose SARS-CoV-2 vaccine, shown in log scale. **(A)** Compared between adult predominantly antibody deficiency (PAD) patients (gray circles; n=62) and pediatric PAD patients (red circles; n=13). **(B)** Subset by PAD disease severity, including adult PAD patients (gray circles; mild: n=12, moderate: n=21, severe: n=19, and secondary: n=10) and pediatric PAD patients (red circles; mild: n=5, moderate: n=3, severe: n=3, and secondary: n=2). Symbols represent unique individuals; bars represent geometric means ( ± 95% confidence intervals) of total indicated patients (n) respectively. *p<0.05, ***p<0.0001.

To investigate SARS-CoV-2 vaccine responses in pediatric PAD patients, we compared the pediatric cohort of PAD patients with anti-spike antibody levels measured after their initial series (n=13) to our previously published adult PAD cohort (n=62) (13). Pediatric patients had a higher mean anti-spike SARS-CoV-2 antibody level compared to adults (2,890.7 vs. 140.1 U/mL; p<0.0001) after two doses of SARS-COV-2 vaccine. When stratified by disease severity, children with severe PAD had significantly higher mean anti-spike antibody levels when compared to adults with severe PAD (2,133.1 vs. 35.7 U/mL; p=0.04). Children with mild, moderate, and secondary PAD trended higher than their adult counterparts, but those differences did not reach statistical significance (mild: 4,285.9 vs. 2,002.5 U/mL; p=0.37, moderate: 4,920.8 vs. 321.8 U/mL; p=0.14, secondary: 767.1 vs. 13.4 U/mL; p=0.15) (**Figure 1**).

Age-related declines in humoral immune function have been widely described (26–32). We hypothesized that the lower anti-spike antibody levels observed in adults compared to children with severe PAD might be driven by underlying higher risk immunophenotypes in the adult patients. To investigate this hypothesis, we compared the immunophenotypes of pediatric and adult PAD patients classified as having severe PAD and found that pediatric patients had higher absolute counts of CD3+ T-cells (1,688.9 vs. 848.3 cells/uL; p=0.05), CD4+ T-cells (918.2 vs. 402.8 cells/uL; p=0.04), CD19+ B-cells (432.6 vs. 74.3 cells/uL; p=0.0004), and CD19+CD27+IgM-IgD- class-switched memory B-cells (16.3 vs. 2.0 cells/uL; p=0.002) (**Supplemental Table 1**). These data were consistent with higher risk immunophenotypes in the severe adult compared to the severe pediatric PAD patients, aligning with the lower SARS-CoV-2 vaccine responses observed in the severe adult PAD patients.

### Risk for low SARS-CoV-2 vaccine response among pediatric PAD patients

**Figure 2:**
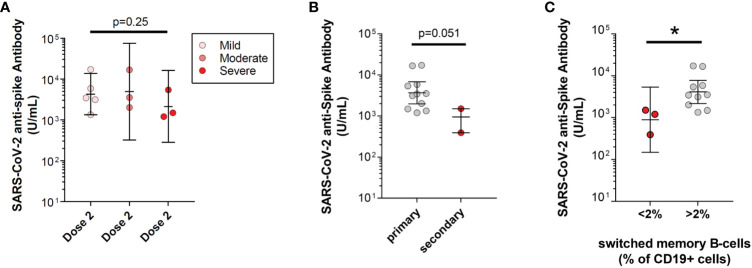
Risk for low SARS-CoV-2 vaccine response in pediatric predominantly antibody deficiency (PAD) patients. SARS-CoV-2 anti-spike antibody levels (U/mL) following initial series two-dose SARS-CoV-2 vaccine, shown in log scale. **(A)** Subset by pediatric PAD disease severity, including mild PAD patients (pink circles; n=5), moderate PAD patients (orange circles; n=3), and severe PAD patients (red circle; n=3). **(B)** Subset by primary PAD patients (gray circles; n=10) and secondary PAD patients (red circles; n=2). **(C)** Subset by immunophenotype, including patients with low class-switched memory B-cells (CD27+IgM-IgD- B-cells <2% of total CD19+ B-cells; red circles; n=3) and high class-switched memory B-cells (CD27+IgM-IgD- B-cells >2% of total CD19+ B-cells; gray circles; n=10). Symbols represent unique individuals, bars represent geometric means ( ± 95% confidence intervals) of total indicated patients (n) respectively. ∗p<0.05.

Previously, severity of PAD, secondary PAD, and a high risk underlying immunophenotype, specifically <2% class-switched memory B-cells, were all shown to be risk factors among PAD adults for lower mean anti-spike antibody levels following SARS-CoV-2 vaccination (13). We sought to understand if these factors also associated with lower antibody responses to SARS-CoV-2 initial series two-dose vaccine among pediatric PAD patients. In contrast to the adults, we did not identify significant differences in anti-spike antibody levels in pediatric patients with severe PAD compared with pediatric patients with moderate PAD and pediatric patients with mild PAD, although severe PAD patients did trend towards lower anti-spike antibody levels (severe: 2,133.1 U/mL, moderate: 4,920.8 U/mL, mild: 4,285.9 U/mL; p=0.25) (**Supplemental Table 2**). Similar to the adults, pediatric PAD patients with low class-switched memory B-cells (defined as <2% of total CD19+ cells) did have lower mean anti-spike antibody levels compared to those without low class-switched memory B-cells (891 vs. 4,114.8 U/mL; p=0.02). Finally, pediatric patients with secondary PAD had a strong trend toward a lower mean anti-spike antibody response after two-dose vaccine series compared to primary PAD patients (767.1 vs. 3,679.2 U/mL; p=0.051) (**Figure 2**).

### Response to third-dose monovalent SARS-CoV-2 vaccine in pediatric PAD patients

**Figure 3:**
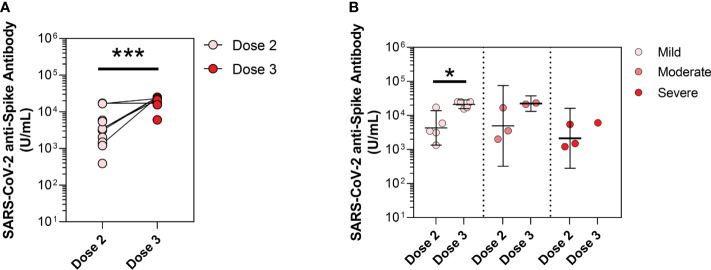
Mean anti-spike antibody levels in pediatric predominantly antibody deficiency (PAD) patients following dose 2 and dose 3 vaccine timepoints. SARS-CoV-2 anti-spike antibody levels (U/mL), shown in log scale. **(A)** At vaccine timepoints post-dose 2 (pink circles, n=13) and post-dose 3 (red circles; n=8). **(B)** Subset by PAD disease severity, including mild PAD patients (dose 2: pink circles; n=5, dose 3: pink circles; n=5), moderate PAD patients (dose 2: orange circles; n=3, dose 3: orange circles; n=2), and severe PAD patients (dose 2: red circles; n=3, dose 3: red circles; n=1). Symbols represent unique individuals, lines represent change in patients drawn at both timepoints, bars represent geometric means ( ± 95% confidence intervals) of total indicated patients (n) respectively. ∗p<0.05, ***p<0.0001.

PAD adults have a significant increase in anti-spike antibody levels following a third monovalent dose of SARS-CoV-2 vaccine (13). We hypothesized that third-dose SARS-CoV-2 monovalent vaccine would similarly increase anti-spike antibody levels in pediatric PAD patients. Among pediatric PAD patients, the mean anti-spike antibody level after initial two-dose series of vaccine was 2,890.7 U/mL, and this increased to 18,267.2 U/mL after the third-dose booster (p<0.0001). This significance held by pairwise comparison for patients in our cohort whose anti-spike antibody levels were measured sequentially after completing the two-dose series and a third-dose vaccine (n=6; mean anti-spike antibody level 4,942.8 to 22,558.6 U/mL; p<0.0001). When stratified by disease severity, we found that mean anti-spike antibody levels increased significantly in pediatric patients with mild PAD before compared to after third-dose immunization (4,285.9 to 21,078.7 U/mL; p=0.02), while patients with moderate PAD showed a strong trend toward an increased response (4,920.8 to 22,188.3 U/mL; p=0.16) (**Figure 3**). We were unable to perform this analysis for severe and secondary PAD patients due to limited data following third-dose immunization for these patients.

### Confounder analysis

To determine whether passive antibody transfer could be confounding the anti-spike antibody analysis, we compared anti-spike antibody levels between patients that did (n=8) or did not (n=7) receive immunoglobulin replacement therapy (IgRT). After a second dose of SARS-CoV-2 vaccine, there was no significant difference in anti-spike antibody levels between pediatric patients who did or did not receive IgRT (2,888.6 vs. 2,892.0 U/mL; p=0.99). After a third-dose of SARS-CoV-2 vaccine, anti-spike antibody levels in patients receiving IgRT trended lower than those that did not receive IgRT, although this did not reach statistical significance (13,893.5 vs. 24,017.6 U/mL; p=0.15) (**Supplemental Figure 2**). Given the potential that this trend may be due, in part, to the more severe immunophenotypes of patients who require IgRT, we further evaluated the underlying immunophenotypes of these patients. PAD patients in our cohort who were receiving IgRT had lower absolute counts of CD3+ T-cell (1,348.3 vs. 2,207.7 cells/uL; p=0.044) and CD8+ T-cell (443.1 vs. 781.9 cells/uL; p=0.043) as compared to those PAD patients not receiving IgRT. There was no significant difference in absolute counts of CD4+ T-cells, CD19+ B-cells, or class-switched memory B-cell (CD19+CD27+IgM-IgD-) (**Supplemental Table 3**).

Given the potential that naturally acquired COVID-19 infections could confound the anti-spike antibody analysis, we compared anti-spike antibody levels of patients with COVID-19 infection before their third-dose antibody level (n=3) to those who had never had a COVID-19 infection at the time of their third-dose blood draw (n=5), and found no statistically significant difference in mean anti-spike antibody levels (22,042 vs. 16,321 U/mL; p=0.44) (**Supplemental Figure 2**).

### Infection outcomes

Eight pediatric patients (53.3%) had naturally acquired SARS-CoV-2 infections during the study. Two patients recovered without treatment. Six patients received either anti-viral (n=4) or corticosteroid (n=2) treatment for their infections. Three patients who received anti-viral therapy received remdesivir, and one patient received ritonavir-boosted nirmatrelvir. There was no rebound COVID-19 in the patient who received ritonavir-boosted nirmatrelvir. No patients died from COVID-19 infection. There were no urgent care visits, ED visits, or hospitalizations in our cohort associated with COVID-19 infection. There were no secondary infections associated with COVID-19 infection.

## Discussion

To our knowledge, this is the first study to assess the efficacy of the SARS-CoV-2 vaccine two-dose series and third additional dose vaccine in pediatric patients ages 4-16 years with PAD. In comparison to adult PAD patients, pediatric PAD patients had significantly higher mean anti-spike antibody levels in response to SARS-CoV-2 vaccination. This may be due, in part, to the composition of the cohorts, with the pediatric cohort being enriched for mild disease and the adult cohort being enriched for moderate and severe disease. However, even when stratified to severe PAD, pediatric patients had significantly higher mean anti-spike antibody levels compared to adult patients. These data were consistent with lower risk immunophenotypic markers in the severe pediatric PAD cohort, including higher absolute counts of CD3+ T-cells, CD4+ T-cells, CD19+ B-cells, and CD19+CD27+IgM-IgD- class-switched memory B-cells.

The anti-spike antibody levels increased significantly after a third monovalent vaccine dose in pediatric PAD patients. This aligns with prior findings in adult PAD patients and prior CDC guidelines recommending three monovalent mRNA SARS-CoV-2 vaccines in pediatric patients (13, 33). Current CDC guidelines have shifted to a three-part series including bivalent vaccine doses in pediatric patients (33). We did not find a significant difference in anti-spike antibody levels by disease severity in pediatric primary PAD. However, certain immunophenotypic markers correlated with a lower anti-spike antibody response in pediatric PAD; specifically, anti-spike antibody levels were lowest in patients with low class-switched memory (CD27+IgM-IgD-) B-cells. This may help clinicians stratify pediatric patients with PAD in terms of identifying those who may be at risk for a lower antibody response after SARS-CoV-2 immunization.

In our study, eight patients had naturally acquired SARS-CoV-2 infection. There were no deaths, and patients did not require ED visits or hospitalization for severe infection. We did not find a statistically significant difference in mean anti-spike antibody levels in those who did or did not have a COVID-19 infection. These data suggest limited confounding by naturally acquired COVID-19 infection to our SARS-CoV-2 vaccine response analysis. Additionally, we did not find a significant difference in anti-spike antibody levels between those receiving IgRT or not, suggesting that there is not a significant effect from passive antibody transfer from immunoglobulin replacement. This highlights the importance of vaccination, even in patients receiving immunoglobulin replacement.

This work is subject to several limitations. The sample size was small, which limits the power of our analysis. In addition, there may be sampling bias in that differences may have existed between patients who consented to be a part of this study and those who did not. This analysis was performed using data from a large but single health care system, so these findings may not be generalizable to other settings. Our cohort was predominantly male and non-Hispanic white, which may also limit generalizability. We analyzed immunogenicity using the Roche Elecsys Anti-SARS-CoV-2 S-antibody test, which may not be generalizable to other assay testing platforms. We were unable to assess anti-spike antibody levels prior to initial vaccination in our cohort, which limits our ability to analyze seroconversion following initial series immunization. There was a difference in dose-to-draw times between dose 2 (85 days) and dose 3 (119 days) in the pediatric PAD cohort, which could skew towards relatively lower anti-spike antibody levels at the post-dose 3 timepoint as compared to the post-dose 2 timepoint. Despite this potential skewing to the null hypothesis, we still observed a significant boost in SARS-CoV-2 anti-spike antibody levels between dose 2 and dose 3 timepoints. Finally, we did not sub-stratify the anti-spike antibody analysis by isotype (IgG, IgA, IgM), as this isotype analysis was comparable when evaluated for the adult PAD cohort (13), which could limit our understanding of the immunogenicity of the SARS-CoV-2 vaccine in pediatric PAD.

Additional studies are needed in larger cohorts of patients to further understand the immune response to SARS-CoV-2 vaccination in pediatric PAD patients, including assessing the benefits of the bivalent vaccine and/or future vaccine platforms. In addition to the antibody responses analyzed here, T-cell response to vaccination provides important cellular immune protection against severe infection, which we are unable to assess with these serologic data and future research regarding this response would be beneficial. Longitudinal studies are needed to evaluate the duration of response to vaccine to determine the optimal vaccination strategy, given that antibody responses can wane over time.

In conclusion, pediatric PAD patients, specifically those with severe PAD, had significantly higher antibody response to SARS-CoV-2 vaccination when compared to adults with severe PAD. Pediatric PAD patients with low (<2% of total CD19+) class-switched memory B-cells had significantly lower response to SARS-CoV-2 vaccination compared to those without. Anti-spike antibody levels increased significantly after receiving a third monovalent SARS-CoV-2 vaccine. These data support CDC guidelines regarding three-dose SARS-CoV-2 vaccine series, including in the pediatric PAD patient demographic.

## Data availability statement

The original contributions presented in the study are included in the article/**Supplementary Materials**, further inquiries can be directed to the corresponding author/s.

## Ethics statement

The studies involving human participants were reviewed and approved by Mass General Brigham IRB. Written informed consent to participate in this study was provided by the participants’ legal guardian/next of kin.

## Author contributions

JRF and SB conceived of the project. DD provided expertise in immunology. MT performed chart review, and wrote the manuscript with aid from JRF and SB. ZB performed statistics herein described. JRF, SB, DD, PH, and CR contributed patients.
