# Supplementary material for: Response to SARS-CoV-2 initial series and additional dose vaccine in pediatric patients with predominantly antibody deficiency
Source: Front Immunol. 2023 Jul 27;14:1217718. doi: 10.3389/fimmu.2023.1217718 (PMC10413262; doi:10.3389/fimmu.2023.1217718)
Supplement: Supplementary file 1 [file DataSheet_1.docx]

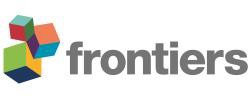


***Supplementary Material***

**Response to SARS-CoV-2 Initial Series and Additional Dose Vaccine in Pediatric Patients with Predominantly Antibody Deficiency**

Tandon, Megha, BA^1*^, DiGiacomo, Daniel V, MD, MPH^1,2^, Zhou, Baijun, MHS^1^, Hesterberg, Paul, MD^1,2^, Rosenberg, Chen E, MD^1,2^, Barmettler, Sara, MD^1,2^ †, Farmer, Jocelyn R, MD, PhD^2,3^†

^1^ Division of Rheumatology, Allergy and Immunology, Department of Medicine, Massachusetts General Hospital, Boston, MA, USA

^2^ Harvard Medical School, Boston, MA, USA

^3^ Division of Allergy and Inflammation, Beth Israel Lahey Health, Boston, Massachusetts, USA

*** Correspondence:** Corresponding Author [megha.tandon@tufts.edu](mailto:megha.tandon@tufts.edu)

† These authors contributed equally to this work and share senior authorship

Supplemental Figures

# Supplemental Figure 1. Timeline of study events

Supplemental Figure 1. Infection, vaccination, and blood draw timepoints denoted by red, green, and blue text, respectively. Total indicated patients (n).


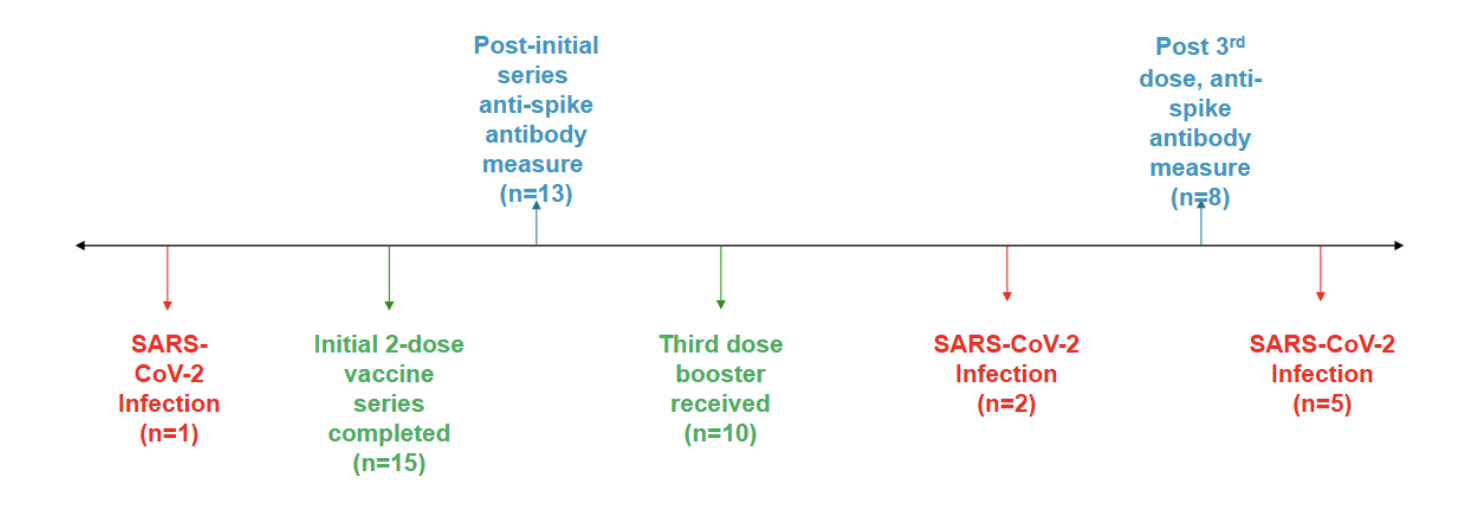


**Supplemental Table 1. Immunophenotype in pediatric and adult predominantly antibody deficiency (PAD) patients by disease severity**

| **Mild PAD** | | | | | |
| --- | --- | --- | --- | --- | --- |
|  | Pediatric | Mean | Adult | Mean | p-value |
|  | n |  | n |  |  |
| CD3+ T-cells (cells/uL) | 6 | 1735.51 | 10 | 1372.51 | 0.319 |
| CD4+ T-cells (cells/uL) | 6 | 952.64 | 10 | 940.41 | 0.948 |
| CD8+ T-cells (cells/uL) | 6 | 602.41 | 10 | 342.30 | 0.090 |
| CD19+ B-cells (cells/uL) | 6 | 418.32 | 10 | 277.49 | 0.2584 |
| CD19+CD27+IgM-IgD-  switched memory B-cells (cells/uL) | 6 | 17.23 | 10 | 9.28 | 0.328 |
| IgG, native level (mg/dL) | 6 | 697.55 | 11 | 678.92 | 0.885 |
| IgA (mg/dL) | 6 | 43.19 | 9 | 113.52 | 0.105 |
| IgM (mg/dL) | 6 | 48.21 | 9 | 58.64 | 0.528 |
| **Moderate PAD** | | | | | |
|  | Pediatric | Mean | Adult | Mean | p-value |
|  | n |  | n |  |  |
| CD3+ T-cells (cells/uL) | 3 | 1657.81 | 20 | 1093.51 | 0.127 |
| CD4+ T-cells (cells/uL) | 3 | 998.52 | 20 | 725.00 | 0.324 |
| CD8+ T-cells (cells/uL) | 3 | 550.59 | 20 | 267.52 | 0.070 |
| CD19+ B-cells (cells/uL) | 3 | 400.78 | 20 | 192.38 | 0.0627 |
| CD19+CD27+IgM-IgD-  switched memory B-cells (cells/uL) | 3 | 16.49 | 16 | 5.20 | 0.086 |
| IgG, native level (mg/dL) | 3 | 665.02 | 17 | 426.30 | 0.173 |
| IgA (mg/dL) | 3 | 28.57 | 17 | 46.82 | 0.478 |
| IgM (mg/dL) | 3 | 28.09 | 17 | 21.32 | 0.599 |
| **Severe PAD** |  |  |  |  |  |
|  | Pediatric | Mean | Adult | Mean | p-value |
|  | n |  | n |  |  |
| CD3+ T-cells (cells/uL) | 4 | 1688.91 | 18 | 848.31 | **0.053** |
| CD4+ T-cells (cells/uL) | 4 | 918.22 | 18 | 402.77 | **0.040** |
| CD8+ T-cells (cells/uL) | 4 | 565.17 | 18 | 350.77 | 0.251 |
| CD19+ B-cells (cells/uL) | 4 | 432.62 | 18 | 74.31 | **0.0004** |
| CD19+CD27+IgM-IgD-  switched memory B-cells (cells/uL) | 4 | 16.31 | 17 | 2.04 | **0.002** |
| IgG, native level (mg/dL) | 3 | 278.07 | 15 | 468.83 | 0.249 |
| IgA (mg/dL) | 3 | 25.53 | 15 | 16.39 | 0.539 |
| IgM (mg/dL) | 3 | 38.99 | 15 | 67.56 | 0.585 |

# Supplemental Table 2. SARS-CoV-2 anti-spike antibody levels in pediatric PAD patients by disease severity

|  | | POST DOSE 2  Geometric mean anti-spike antibody (U/mL)  (n=13) | | p-value | | POST DOSE 3  Geometric mean anti-spike antibody (U/mL)  (n=8) | | p-value | |
| --- | --- | --- | --- | --- | --- | --- | --- | --- | --- |
| Mild | IgG Subclass Deficiency | 4285.9 | 3030.3 | 0.53 | 21078.7 | | 25000 | -- |  |
|  | SAD |  | 17151 |  |  |  | 17132 |  |  |
|  | Primary Hypogammaglobulinemia |  | -- |  |  |  | 15545 |  |  |
| Moderate | CVID | 4920.8 | 4920.8 |  | 22188.3 | | 22188.3 |  |  |
| Severe | Complicated PAD | 2133.1 | 2133.1 |  | 6052 | | 6052 |  |  |

| Primary PAD | 3679.24 | 0.05 | -- | -- |
| --- | --- | --- | --- | --- |
| Secondary PAD | 767.11 |  | -- |  |

*PAD,* Predominant Antibody Deficiency*; SAD,* Specific Antibody Deficiency*; CVID,* Common Variable Immune Deficiency

# Supplemental Figure 2. Mean anti-spike antibody levels in pediatric predominantly antibody deficiency (PAD) patients by immunoglobulin replacement use and COVID-19 infection status

Supplemental Figure 2. SARS-CoV-2 anti-spike antibody levels (U/mL) in pediatric PAD patients, shown in log scale. (A) Compared between patients receiving immunoglobulin replacement therapy (IgRT) (blue circles; n=6 following dose 2 immunization, n=4 following dose 3 immunization) and patients not receiving immunoglobulin replacement therapy (no IgRT) (green circles; n=7 following dose 2 immunization, n=4 following dose 3 immunization). (B) Compared between patients with prior history of PCR-confirmed COVID-19 infection (orange circles; n=2 following dose 3 immunization) and no prior history of COVID-19 infection (blue circles; n=5 following dose 3 immunization). Symbols represent unique individuals and bars represent geometric means (±95% confidence intervals) of total indicated patients (n). ns=not significant.

**A B**

#
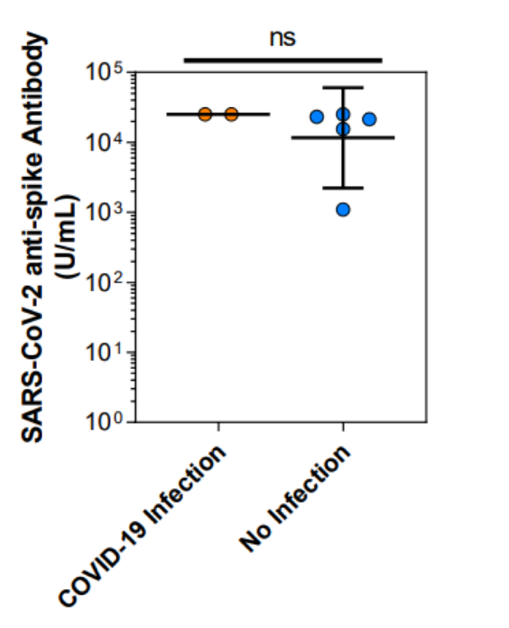

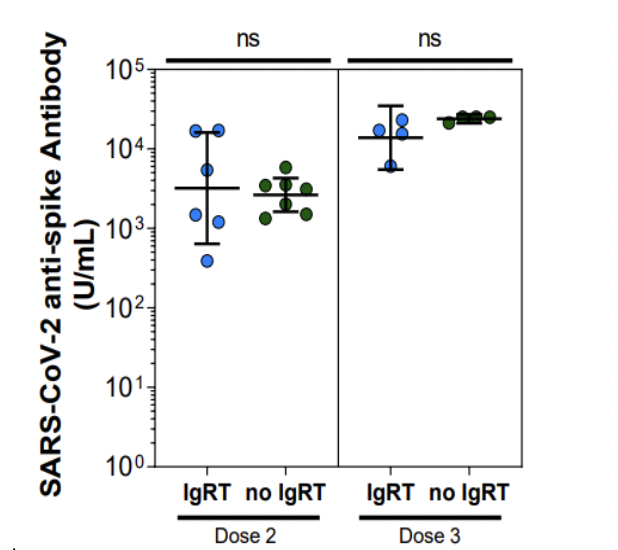


**Supplemental Table 3. Immunophenotype in pediatric predominantly antibody deficiency (PAD) patients by immunoglobulin replacement therapy (IgRT) status**

|  | OFF IgRT Therapy | | ON IgRT Therapy | | p-value |
| --- | --- | --- | --- | --- | --- |
|  | n | Mean | n | Mean |  |
| CD3+ T-cells (cells/uL) | **7** | 2207.68 | 8 | 1348.33 | **0.044** |
| CD4+ T-cells (cells/uL) | 7 | 1225.31 | 8 | 753.96 | 0.062 |
| CD8+ T-cells (cells/uL) | 7 | 781.88 | 8 | 443.14 | **0.043** |
| CD19+ B-cells (cells/uL) | 7 | 435.58 | 8 | 361.65 | 0.520 |
| CD19+CD27+IgM-IgD-  switched memory B-cells (cells/uL) | 7 | 20.63 | 8 | 9.53 | 0.183 |
